# Chemical composition, antibiofilm, cytotoxic, and anti-acetylcholinesterase activities of *Myrtus communis* L. leaves essential oil

**DOI:** 10.1186/s12906-022-03583-4

**Published:** 2022-05-20

**Authors:** Lucia Caputo, Francesca Capozzolo, Giuseppe Amato, Vincenzo De Feo, Florinda Fratianni, Giovanni Vivenzio, Filomena Nazzaro

**Affiliations:** 1 Department of Pharmacy, University of Salerno, via Giovanni Paolo II, 132, 84084 Fisciano, Salerno, Italy; 2 Institute of Food Science, CNR-ISA, via Roma 64, 83100 Avellino, Italy

**Keywords:** *Myrtus communis*, Essential oil, Antibacterial activity, Biofilm, Biofilm metabolism, Cytotoxic activity, SH-SY5Y cells, Anti-acetylcholinesterase activity

## Abstract

**Background:**

The potential of essential oils (EOs) and of their principal constituents for eradication of biofilm and at the same time the research of new potential acetylcholinesterase inhibitors is gaining increasing interest in last years. The aims of this study were to determine the chemical composition and to evaluate the antibacterial, cytotoxic, and anti-acetylcholinesterase properties of *Myrtus communis* leaves essential oil and its main constituents.

**Methods:**

Essential oil was obtained by hydrodistillation of *M. communis* L. leaves and was analyzed by GC and GC–MS. The antimicrobial activity was carried out against both gram-negative and gram-positive bacteria. The microdilution method was used to estimate the minimum inhibitory concentrations (MICs). Then, the capacity of essential oil and its main constituent to inhibit biofilm growth, with the method of O’Toole and Kolterand, and the metabolic activity of biofilm cells through the MTT colorimetric method were evaluated at different times. Moreover, was studied the potential cytotoxic activity against SH-SY5Y cell line with MTT assay and the anti-acetylcholinesterase activity using Ellman’s assay.

**Results:**

Myrtenyl-acetate, 1,8 cineole, α-pinene, and linalool were the main components in the EO. The myrtle EO, at the minimum tested dose (0.4 mg/ml), inhibited *S. aureus* biofilm by 42.1% and was capable of inhibiting the biofilm cell metabolism in all tested strains, except *Staphylococcus aureus.* Moreover, the EO showed good cytotoxic and anti-acetylcholinesterase activities IC_50_ of 209.1 and 32.8 μg/ml, respectively.

**Conclusions:**

The results suggest that myrtle EO and its main constituents could be used as possible products that could act against the resistant pathogenic species *E. coli*, *P. aeruginosa*, *L. monocytogenes* and *S. aureus*, on the other hand, as possible coadjutants in the treatment of neurological diseases.

## Background

Myrtle (*Myrtus communis* L*.*, Myrtaceae) is a plant used worldwide in traditional medicine. It is a spontaneous evergreen shrub or a small tree of Mediterranean area, growing along the coasts, on the islands, and the internal hill [1, 2]. The essential oils (EOs) derived from fresh leaves and/or berries are used as food flavoring (meat, sauces, etc.), for spirits and in the perfume and cosmetic industry [3]. Moreover, in folk medicine, these EOs have an important role in the treatment of gastrointestinal diseases for their astringent proprieties and are used as antimicrobial, antioxidant, and anti-inflammatory agents [4]. Several studies reported that *M. communis* essential oil and its principal components, such as 1,8-cineole, linalool, eugenol, α-terpineol and γ-terpinene, were active against both Gram-positive and Gram-negative bacteria [5–7].

Biofilms are constituted by microbial cells absorbed in a matrix of extracellular polymeric substances. These cells can cause chronic infections, persistent inflammation, and tissue damage [8] and are very different from planktonic cells. In fact, the biofilm is constituted in well-organized hierarchically communities that protect the cells from adverse environmental conditions and antibacterial agents [9, 10].

The potential of EOs and their principal constituents for the eradication of biofilm is gaining increasing interest in recent years as new antibacterial agents, especially for the increasing emergence of bacterial resistance [10, 11]. This phenomenon, as well as the occurrence of the bacterial transformation giving rise to more aggressive bacteria, represent two problems of great impact on human health. Such aspects are encouraging the research toward the identification of new antibacterials, also from natural sources, which can represent alternative and more efficient modes of treatment with respect to the conventional antibacterial drugs, especially when people are faced with particularly aggressive and resistant bacteria. For example, infections by *Staphylococcus aureus* and *Pseudomonas aeruginosa*, mainly through biofilm formation, is a problem of great relevance. These infections, carrying on antigen presentation, lead to chronic inflammation and makes common eradication treatments less effective [12]. Similarly acts the uropathogenic *Escherichia coli*; in fact, its biofilms are problematic to remove from the surface of hospital catheters [13], and *Listeria monocytogenes*, able to infect food products also by the formation of biofilms [14]. Also, plants are subjected to bacterial infection: for example, the biofilm formed by the Gram-negative *Pectobacterium carotovorum* causes soft rot in some food plants due to the release of exo-enzymes and increases bacterial resistance during plant disinfection [15].

Different EOs have already been tested in an attempt to find potential inhibitors of the formation of biofilm [16–18]. Conversely, few studies are reported on the identification of those extracts and/or EOs capable of affecting the metabolism of the cells present in the biofilm organization, which profoundly differ from planktonic cells of the same species due to complex phenotypic and metabolic changes that regulate some cell events, such as adhesion, sporulation, starvation survival, rough-smooth phase variations, etc. This multifaceted scenario also led to differences in the susceptibility of planktonic and biofilm bacterial cells to antimicrobial agents.

Since the 1990s, a very promising field of application of EOs is the study of their cytotoxic properties against several cancer cell lines [19]. Nevertheless, few studies reported the cytotoxicity of the EO of *M. communis* against several cell lines: human cervix adenocarcinoma (HeLa), human breast cancer (MCF-7), *Mus musculus* mastocytoma cells (P815), and human colon colorectal adenocarcinoma (HT29) [20–22]. Until now, no research has been carried out on the possible cytotoxicity of *M. communis* or of any plants belonging from Myrtaceae family against neuroblastoma, the most common tumor among children less than one year of age [23].

Moreover, in the last years, the research of natural substances for therapeutic aims highlights a potential source of acetylcholinesterase (AChE) inhibitors in plants [24]. This interest derives from the traditional use of several plants to treat neurodegenerative diseases [25]. Several EOs and their constituents have been investigated for their inhibition activity of AChE [26], but only one study reported the possible anti-acetylcholinesterase action of myrtle EO [27].

Therefore, the present study was carried out to determine the chemical composition of the EO from leaves of *M. communis* leaves and to investigate on antimicrobial, antibiofilm, cytotoxic, and anti-acetylcholinesterase activities of myrtle EO and its main constituents.

## Materials and methods

### Plant material

Leaves of *M. communis* were collected in Bellosguardo (Salerno, Italy) in February 2020. The plant was identifiedby Prof. Vincenzo De Feo, full professor at University of Salerno . A voucher specimen (DF/2020/314) is stored in the herbarium of the Medical Botany Laboratory, University of Salerno. The use of *M. communis* L. leaves in the present study compiles with international guidelines; permissions or licenses were not necessary to collect this plant species.

### Isolation of essential oil

Fresh leaves were subjected to steam distillation for 3 h according to the standard procedure described by the European Pharmacopoeia [28]. The distillation furnished yellow oil in 0.9 % yield on a dry mass basis. The oil was solubilized in *n*-hexane, filtered over anhydrous sodium sulphate, and stored under N_2_ at +4 °C in dark-sealed vial until analysis.

### GC-FID analysis

Analytical gas chromatography was performed on a Perkin-Elmer Sigma-115 gas chromatograph equipped with a FID and a data handling processor. The separation was achieved using a HP-5 MS fused-silica capillary column (30 m × 0.25 mm i.d., 0.25 µm film thickness). Column temperature: 40 °C, with 5 min initial hold, and then to 270 °C at 2 °C/min, and finally at 270 °C (20 min); injection mode splitless (1 µL of a 1:1000 *n*-hexane solution). Injector and detector temperatures were 250 °C and 290 °C, respectively. Analysis was also run by using a fused silica HP Innowax polyethylene glycol capillary column (50 m × 0.20 mm i.d., 0.25 µm film thickness). In both cases, helium was used as carrier gas (1.0 ml/min) [29].

### GC/MS analysis and identification of constituents

GC/MS analyses were performed on an Agilent 6850 Ser. II apparatus, fitted with a fused silica DB-5 capillary column (30 m × 0.25 mm i.d., 0.33 µm film thickness), coupled to an Agilent Mass Selective Detector MSD 5973; ionization energy voltage 70 eV; electron multiplier voltage energy 2000 V. Mass spectra were acquired in the range 40–500 amu, scan time 5 scans/s. Gas chromatographic conditions were as reported above; transfer line temperature, 295 °C. Most constituents were identified by comparison of their Kovats retention indices (Ri) [calculated in relation to a series of *n*-alkanes (C_10_–C_35_)], with either those of the literature [30–33], by accurate analysis of mass spectra on both columns and by their comparison with those of authentic compounds available in our laboratories by means of NIST 08 and Wiley 275 libraries. The components’ relative concentrations were obtained by peak area normalization.

### Microorganisms and culture conditions

Three Gram negative (*E. coli* DSM 8579, *P. aeruginosa* ATCC 50071, *P. carotovorum* DSM 102074) and two Gram positive (*S. aureus* DMS 25923, *L. monocytogenes* ATCC 7644) bacterial strains, provided by DSMZ (Deutsche Sammlung von Mikroorganismen und Zellkulturen GmbH, Braunschweig, Germany) were used. Bacteria were grown in Luria Bertani (LB) broth (Sigma, Milano, Italy) for 18 h at 37 °C and 80 rpm (Corning LSE, Pisa, Italy). *P. carotovorum* was grown at 28 °C and 80 rpm*.* Bacteria were serially diluted in sterile physiological solution and spread onto Muller-Hinton agar plates. Using an aseptic method, a single colony of each strain was transferred into a 10 ml sterile tube containing the iso-sensitized broth and placed for the growth in incubator for 18 h at 80 rpm (Corning) and different temperatures depending on the strain. Pellet was washed and resuspended in 10 ml sterile saline solution. The optical density was recorded at 600 nm, and serial dilutions were performed to ensure an optical density at 0.5.

### Minimal Inhibitory Concentration (MIC)

The Minimal Inhibitory Concentration (MIC) values were calculated following the method of Saker and coworkers [34] and Fratianni and coworkers [35] on microtiter-plates. A resazurin solution, used as an indicator solution, was prepared by dissolving 270 mg in 40 ml of sterile distilled water. Through the use of a vortex mixer, the indicator was perfectly dissolved so to have a homogeneous solution. The EO, and its main components, myrtenyl acetate, 1,8 cineole, α-pinene, and linalool, were dissolved in sterile DMSO. Two-fold serial dilutions were prepared to obtain 50 μL of EO, myrtenyl acetate, 1,8 cineole, α-pinene, and linalool, in serially descending concentrations in each well. Thirty-five μL of 3.3 × strength iso-sensitized broth and 5 μL of resazurin solution were supplemented, to reach a final volume/well of 240 μL with different volumes of sterile Muller-Hinton broth (Sigma-Aldrich, Milano, Italy) previously set. Lastly, 10 μL of bacterial suspension were added to each well to get a concentration of about 5 × 10^5^cfu/ml, to obtain a final volume of 250 μL. Sterile DMSO and tetracycline (dissolved in DMSO, 1 mg/ml) were used as negative and positive control, respectively. Multiwell plates were prepared in triplicate and incubated at 37 °C for 24 h. The color changes were assessed visually. The lowest concentration at which the color change (from dark purple to colorless) was visualized, indicated the MIC value that thus represented also the lowest concentration of compound giving rise to a marked reduction in the appearance of growth compared to the growth control.

### Biofilm inhibitory activity

The effects of the EO and its main components, myrtenyl acetate, 1.8 cineole, α-pinene, and linalool, at concentrations ranging from 0.4 to 2.0 mg/ml, were evaluated using the method of Fratianni and coworkers [35] using 96-well microtiter plates. In each well, the overnight bacterial cultures were adjusted to 0.5 McFarland with fresh culture broth. Ten µL of the diluted cultures were distributed in each well; then different volumes of the samples and Muller-Hinton broth were added, to reach a final volume of 250 µL/well. Microplates were completely covered with parafilm to avoid the evaporation of samples with relative loss of volume and incubated for 48 h at 37 °C (except *P. carotovorum* that was incubated at 28 °C). Planktonic cells were removed, and the attached cells were gently washed twice with sterile physiological saline. After that, 200 µL of methanol were added to each well, retaining it for 15 min to fix the sessile cells. After discharge of methanol, each plate was left until complete dryness of samples. Staining of the adhered cells was performed by adding 200 µL of 2% *w/v* crystal violet solution to each well. After 20 min, wells were gently washed with the sterile physiological solution and left to dry. Two hundred microliters of glacial acetic acid 20% *w/v* were added to allow the release of the bound dye. The absorbance was measured at OD = 540 nm (Varian Cary Spectrophotometer model 50 MPR, Cernusco sul Naviglio, Italy). The percent value of biofilm inhibition was calculated respect to control (cells grown without the presence of the samples). Triplicate tests were done, and the average results were taken for reproducibility.

### Metabolic activity of biofilm cells

The effects of different concentrations of the myrtle EO, and its main components, myrtenyl acetate, 1.8 cineole, α-pinene and linalool, ranging from 0.4 to 2.0 mg/ml on the metabolic activity of biofilm cells was evaluated through the MTT colorimetric method, using 96-well microtiter plates [35] The overnight bacterial cultures, grown at the due temperatures, were adjusted to 0.5 McFarland and treated as above described. After 48 h incubation, bacterial suspension was removed, and 150 µL of PBS and 30 µL of 0.3% MTT [3-(4,5-dimethylthiazol-2-yl)-2,5-diphenyltetrazolium bromide, Sigma, Milan, Italy] were added, keeping microplates at 37 °C. After 2 h, MTT solution was removed and, after two washing steps with 200 μL of sterile physiological solution, 200 µL of DMSO were added to allow the dissolution of the formazan crystals, which were measured at OD = 570 nm (Varian Cary Spectrophotometer model 50 MPR, Cernusco sul Naviglio, Italy). Triplicate tests were done and the average results were taken for reproducibility.

### MTT assay

Human neuroblastoma (SH-SY5Y) cancer cells purchased from ATCC Bioproducts (ATCC, Manassas,VA, USA) were cultured in Roswell Park Memorial Institute Medium (RPMI) supplemented with 1% L-glutamine, 10% heat-inactivated fetal bovine serum (FBS),1% penicillin/streptomycin (all from Sigma Aldrich) at 37°C in an atmosphere of 95% O_2_ and 5% CO_2_.

Cells were plated (5 × 10^3^) in 96-well culture plates in 150 µL of culture medium and incubated at 37 °C in a humidified atmosphere of 95% O_2_ and 5% CO_2_. The day after, a 150 µL aliquot of serial dilutions of the EO and its main constituents (500–25 µg/ml) was added to the cells and incubated for 24 h. DMSO was used as a control. Cell viability was assessed through MTT (3-(4,5-dimethylthiazol-2-yl)-2,5-diphenyl tetrazolium bromide) assay. Briefly, 30 µL of MTT (5 mg/ml) were added and the cells were incubated for an additional 3 h. After that, cells were lysed, and the dark blue crystals were solubilized with 30 µL of a solution containing 50%, v/v, N,N-dimethylformamide, 20%, w/v, SDS with an adjusted pH of 4.5. The optical density (OD) of each well was measured with a microplate spectrophotometer (Thermo Scientific Multiskan GO, Monza, Italy) equipped with a 520 nm filter. Cell viability in response to treatment was calculated as a percentage of control cells treated with DMSO at the final concentration 0.1% [36].

$$viable cells (\%)=\left[\frac{OD treated cells}{OD control cells}\right]*100$$

### Anti-acetylcholinesterase activity

AChE inhibitory activity assay was performed according to a previously described spectrophotometric method [37] with minor modifications [38]. Briefly, in a total volume of 1 ml, 415 µL of Tris-HCl buffer 0.1 M (pH 8), 10 µL of different concentrations of extract dissolved in methanol, and 25 µL of AChE solution (0.28 U/ml) were incubated for 15 min at room temperature. Seventy-five microliters of a solution of AChI (1.83 mM) and 475 µL of DTNB were added, and the final mixture was incubated for 30 min at room temperature.

Absorbance was measured at 405 nm by a spectrophotometer (Thermo Scientific Multiskan GO, Monza, Italy). Galanthamine was used as a positive control. Bidistilled water, instead of the EO or galanthamine, was used as a negative control. The inhibition rate (%) of AChE activity was calculated by comparison with the negative control by using the following equation:

$$\%=\left[\frac{\left({A}_{C}- {A}_{S }\right)}{{A}_{C}}\right]*100$$

### Statistical analysis

All experiments were carried out in triplicate. Data of antimicrobial, cytotoxic, and anti-acetylcolinesterase activities were statistically analyzed using GraphPad Prism 6.0 software (GraphPad Software Inc., San Diego, CA, United States) followed by the comparison of means (two-way ANOVA) using Dunnett’s multiple comparison test, at the significance level of *p* < 0.05.

## Results

### Phytochemical analyses

**Fig. 1:**
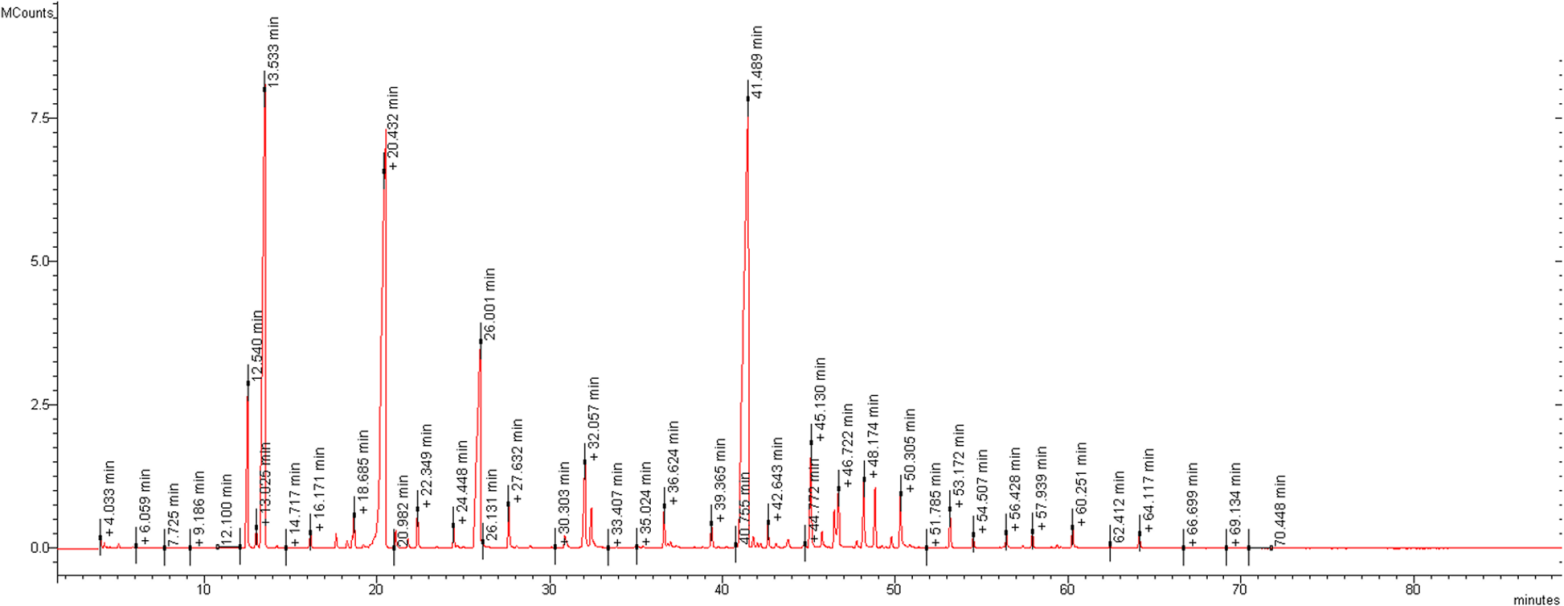
GC–MS chromatogram of the leaves essential oil of *M. communis* L

Hydrodistillation of three samples of the leaves of *M. communis* provided pale-yellow oils in 0.33 ± 0.05% yield on a dry mass basis. The GC profile of essential oil is present in Fig. 1. Table 1 shows the chemical composition of the EO; compounds are listed according to their elution order on a HP-5MS.

**Table 1:** Chemical composition *Myrtus communis* L. leaves essential oil. Results are expressed as mean area percentage (%) ± standard deviation (S.D.) of three independent determinations (*n* = 3)

N	Compounds	%	KI^a^	KI^b^	Identification^c^
**1**	3*Z*- Hexenal	0.1 ± 0.02	744	798	1,2
**2**	2*E*- Hexenal	0.1 ± 0.03	747	855	1,2
**3**	*n*-Nonane	T	752	900	1,2
**4**	Isobutyl isobutyrate	0.1 ± 0.02	758	911	1,2
**5**	Tricyclene	T	849	926	1,2
**6**	Heptyl isobutanoate	3.2 ± 0.3	855	1250	1,2
**7**	α-Thujene	0.4 ± 0.01	861	930	1,2,3
**8**	α-Pinene	14.7 ± 1.2	868	939	1,2,3
**9**	Camphene	T	877	954	1,2,3
**10**	Sabinene	0.3 ± 0.03	902	975	1,2,3
**11**	β-Pinene	0.3 ± 0.04	921	979	1,2,3
**12**	δ-3-Carene	0.3 ± 0.02	929	1011	1,2,3
**13**	β- Myrcene	0.1 ± 0.01	930	990	1,2,3
**14**	Butyl-2-methylbutanoate	0.2 ± 0.01	933	-	1,2
**15**	α-Terpinene	0.1 ± 0.02	941	1017	1,2,3
**16**	1,8-Cineole	21.9 ± 2.3	957	1031	1,2,3
**17**	*E*-β-Ocimene	1.1 ± 0.5	966	1050	1,2
**18**	γ-Terpinene	0.4 ± 0.03	1006	1059	1,2,3
**19**	Terpinolene	0.1 ± 0.02	1008	1088	1,2,3
**20**	dehydro-Linalool	T	1013	1090	1,2
**21**	Linalool	9.1 ± 1.6	1026	1096	1,2,3
**22**	Myrcenol	0.2 ± 0.03	1028	1122	1,2
**23**	*cis*-*p*-Menth-2-*n*-1-ol	0.1 ± 0.02	1033	1121	1,2
**24**	*allo* Ocimene	0.8 ± 0.04	1048	1132	1,2
**25**	*trans*-Pinocarveol	0.1 ± 0.01	1054	1139	1,2
**26**	3E-6Z-Nonadienol	0.1 ± 0.03	1065	1153	1,2
**27**	*neo*-Menthol	T	1082	1165	1,2
**28**	δ-Terpineol	T	1084	1166	1,2,3
**29**	Terpinen-4-ol	0.4 ± 0.05	1092	1177	1,2,3
**30**	α-Terpineol	2.3 ± 0.4	1101	1188	1,2,3
**31**	Myrtenal	0.1 ± 0.04	1103	1195	1,2,3
**32**	Myrtenol	0.8 ± 0.03	1106	1195	1,2
**33**	Methyl chavicol	0.2 ± 0.05	1108	1196	1,2
**34**	Iso-dihydro-carveol	T	1127	1214	1,2
**35**	Fraganol	0.1 ± 0.02	1144	1215	1,2
**36**	*cis*-Myrtanol	T	1160	1253	1,2
**37**	Linalool acetate	0.8 ± 0.06	1167	1257	1,2
**38**	δ-Octalactone	T	1177	1278	1,2
**39**	Isobornyl acetate	T	1191	1285	1,2
**40**	*trans*-Pinocarvyl acetate	0.6 ± 0.03	1199	1298	1,2
**41**	Carvacrol	0.1 ± 0.02	1214	1299	1,2,3
**42**	Myrtenyl acetate	29.8 ± 2.4	1236	1326	1,2
**43**	*iso*-dihydro-Carveol acetate	0.3 ± 0.02	1237	1329	1,2
**44**	Carvyl acetate	0.1 ± 0.03	1241	1342	1,2
**45**	α-Terpinyl acetate	0.5 ± 0.04	1250	1349	1,2
**46**	Citronellyl acetate	0.1 ± 0.02	1257	1352	1,2
**47**	Geranyl acetate	2.6 ± 0.5	1268	1381	1,2,3
**48**	Methyl eugenol	0.9 ± 0.02	1302	1403	1,2
**49**	*Z*-Caryophyllene	1.3 ± 0.06	1306	1408	1,2,3
**50**	γ-Elemene	0.1 ± 0.01	1323	1436	1,2,3
**51**	α-Humulene	1.1 ± 0.02	1340	1454	1,2,3
**52**	*p*-Menth(1,8 dien)-9-ol	0.4 ± 0.02	1343	1374	1,2,3
**53**	Bisabolol	0.2 ± 0.08	1347	1685	1,2,3
**54**	Thymohydro quinone	0.7 ± 0.06	1406	1555	1,2
**55**	Flavesone	0.2 ± 0.02	1428	1547	1,2
**56**	Caryophyllene oxide	0.3 ± 0.02	1461	1583	1,2,3
**57**	Humulene epoxide II	0.3 ± 0.01	1486	1608	1,2
**58**	*allo*-Aromadendrene epoxide	0.1 ± 0.02	1504	1641	1,2
**59**	*n*-Octadecanol	0.5 ± 0.06	1520	2077	1,2
	**Total**	**98.4**			
	**Monoterpenes hydrocarbons**	**18.6**			
	**Oxygenated monoterpenes**	**71.7**			
	**Sesquiterpenes hydrocarbons**	**2.6**			
	**Oxygenated sesquiterpenes**	**1.4**			
	**Other**	**4.1**			

^a, b^ are the Kovats retention indices determined relative to a series of *n*-alkanes (C10–C35) on the apolar HP-5 MS and the polar HP Innowax capillary columns, respectively; ^c^ identification method: 1 = comparison of the Kovats retention indices with published data; 2 = comparison of mass spectra with those listed in the NIST 02 and Wiley 275 libraries and with published data; 3 = coinjection with authentic compounds; c -: not detected; t = trace (< 0.05%)

Altogether, 59 compounds were identified, accounting for 98.4% of the total oil. Oxygenated monoterpenes were the main components (71.7%), followed by monoterpenes hydrocarbons (18.6%) and sesquiterpenes hydrocarbons (2.6%). Myrtenyl-acetate (29.8%), 1,8-cineole (21.9%), α-pinene (14.7%) and linalool (9.1%) were the main constituents. Other compounds, in a lesser amount, were heptyl isobutanoate (3.2%), geranyl-acetate (2.6%), α-terpineol (2.3%), (*Z*)-caryophyllene (1.3%) and α-humulene (1.1%).

### Antibacterial activity

Through the resazurin test, the minimal inhibitory concentration (MIC) necessary to block the growth of the bacteria used as tester strains was evaluated. Results are shown in Table 2.

**Table 2:** MIC exhibited by the myrtle EO and its main components against the pathogenic strains

	**Myrtle EO** **(mg/ml)**	**Myrtenyl acetate** **(mg/ml)**	**1,8-Cineole** **(mg/ml)**	**α-pinene** **(mg/ml)**	**Linalool** **(mg/ml)**	**Tetracycline** **(**μ**g/ml)**
*E*. *coli*	6 (± 0.2)	7 (± 0.2)^***^	7 (± 0.2)^***^	7 (± 0.2)^***^	6 (± 0.2)	18 (± 0.1)
*L*. *monocytogenes*	3 (± 0.2)	6 (± 0.2)^****^	4 (± 0.1)^***^	6 (± 0.2)^****^	6 (± 0.2)^****^	24 (± 0.1)
*P*. *carotovorum*	4 (± 0.5)	7 (± 0.2)^****^	> 10	5 (± 0.2)^**^	5 (± 0.2)^**^	14 (± 0.1)
*P*. *aeruginosa*	3 (± 0.5)	4 (± 0.1)^*^	> 10	8 (± 0.5)^****^	4 (± 0.2)^*^	25 (± 0.2)
*S*. *aureus*	5 (± 0.1)	8 (± 0.5)^****^	4 (± 0.2)^**^	4 (± 0.2)^**^	6 (± 0.2)^**^	8 (± 0.2)

The data, expressed in mg/ml, are the average of three independent experiments (± SD). *p < 0.05, **p < 0.01; ***p < 0.001; ****p < 0.0001 compared with values obtained with EO (ANOVA followed by Dunnett’s multiple comparison test)

The whole EO exhibited an effective inhibitory activity that did never exceed 6 mg/ml (in *E. coli*). The four main components of the EO showed a MIC higher than of the EO, and in some cases, values reach up 10 mg/ml. The whole EO perhaps could act as an antibacterial agent due to the synergistic action of more components that, considered individually, did not exhibit the same potency.

The capacity of the EOs to block or limit the formation of biofilm (through the use of the crystal violet assay) and the effect of the EOs on the bacterial cell metabolism (observed by the MTT test) were also evaluated. Results are shown in Tables 3 and 4, respectively.

The addition of the different doses (final concentration 0.4, 1, and 2 mg/ml, calculated after the MIC analysis), was carried out at three different times: at zero time before the formation of the biofilm (to evaluate the ability of the EO and its main components to block the formation of biofilm *ab origine*); after 24 h growth (to evaluate the capacity to act on mature biofilm) and after 48 h growth (when the biofilm is extremely mature); therefore, in the presence of different metabolic and microbial cell growth conditions (Table 3).

**Table 3:** Percent inhibition of the formation of biofilm exhibited by myrtle EO and its main components

Time	Myrtle EO (mg/ml)			Myrtenyl acetate (mg/ml)			1,8-Cineole (mg/ml)			α-Pinene (mg/ml)			Linalool (mg/ml)		
0	**0.4**	**1**	**2**	**0.4**	**1**	**2**	**0.4**	**1**	**2**	**0.4**	**1**	**2**	**0.4**	**1**	**2**
EC	7.20 (± 1.05)	13.9 (± 1.33)	26.25 (± 1.67)	0 (± 0)	2.01 (± 0.02)	3.45 (± 0.05)	0 (± 0)	6.11 (± 0.57)	13.75 (± 0.57)	0 (± 0)	6.19 (± 0.57)	20.76 (± 0.57)	0 (± 0)	3.12 (± 0.16)	33.68 (± 0.44)^****^
LM	2.03 (± 0.03)	34.79 (± 1.33)	64.55 (± 1.15)	0 (± 0)	2.97 (± 0.03)	39.03 (± 1.67)	0 (± 0.0)	0 (± 0)	17.7 (± 1.67)	17.72 (± 1.56)^****^	29.83 (± 1.67)	44.49 (± 1.67)	0 (± 0)	5.44 (± 0.04)	7.12 (± 0.08)
PC	0 (± 0)	44.27 (± 1.57)	47.28 (± 1.57)	0 (± 0)	0 (± 0)	2.08 (± 0.09)	0 (± 0)	0 (± 0)	36.81 (± 1.67)	0 (± 0)	0 (± 0)	39.65 (± 2.25)	0 (± 0)	36.95 (± 1.12)	55.46 (± 1.44^****^)
PA	0 (± 0)	3.33 (± 0.57)	66.67 (± 1.33)	14.11 (± 0.89)^****^	57.46 (± 1.33)^****^	57.86 (± 2.13)	0 (± 0)	0 (± 0)	40.22 (± 1.57)	20.44 (± 2.26)^****^	33.32 (± 1.57)^****^	77.45 (± 2.33)^****^	0 (± 0)	16.91 (± 0.97)^****^	17.57 (± 0.67)
SA	42.1 (± 1.57)	43.55 (± 1.25)	56.7 (± 1.33)	0 (± 0)	54.54 (± 2.67) ^****^	49.08 (± 2.33)	0 (± 0.0)	32.03 (± 2.33)	59.41 (± 1.67)	26.23 (± 1.56)	28.07 (± 2.16)	91.39 (± 1.67)^****^	0 (± 0)	22.50 (± 1.57)	23.07 (± 1.43)
24 h															
EC	0 (± 0)	0 (± 0)	0 (± 0)	0 (± 0)	0 (± 0)	0 (± 0)	0 (± 0)	0 (± 0)	0 (± 0)	0 (± 0)	0 (± 0)	0 (± 0)	0 (± 0)	0 (± 0)	0 (± 0)
LM	0 (± 0)	13.65 (± 1.25)	15.93 (± 1.24)	0 (± 0)	0 (± 0)	0 (± 0)	0 (± 0)	0 (± 0)	0 (± 0)	7.14 (± 1.67)	18.48 (± 1.67)	28.99 (± 1.57)	0 (± 0)	9.2 (± 0.04)	44.13 (± 1.14)
PC	0 (± 0)	0 (± 0)	2.8 (± 1.57)	0 (± 0)	0 (± 0)	0 (± 0)	0 (± 0)	0 (± 0)	2.51 (± 0.16)	0 (± 0)	0 (± 0)	2.59 (± 1.67)	0 (± 0)	0 (± 0)	5.37 (± 1.67)
PA	0 (± 0)	0 (± 0.57)	4.97 (± 1.33)	0 (± 0)	0 (± 0)	6.33 (± 0.57)	0 (± 0)	0 (± 0)	16.61 (± 1.57)	0 (± 0)	0 (± 0)	14.66 (± 2.33)^****^	0 (± 0)	5.43 (± 0.57)	7.07 (± 0.67)
SA	0 (± 0)	0 (± 0)	1.1 (± 0.02)	12.34 (± 0)	19.92 (± 1.44)	50.62 (± 1.67)	0 (± 0)	9.82 (± 0.57)	18.09 (± 1.13)	0 (± 0)	0 (± 0)	0 (± 0)	0 (± 0)	0 (± 0)	35.68 (± 1.43)
48 h															
EC	12.24 (± 1.13)	32.07 (± 1.67)	42.49 (± 2.24)	14.07 (± 0.57)	50.42 (± 1.14)	52.15 (± 2.25)	23.56 (± 1.67)	0 (± 0)	0 (± 0)	0 (± 0)	0 (± 0)	0 (± 0)	15.67 (± 1.07)	49.51 (± 1.23)	50.45 (± 1.15)
LM	0 (± 0)	0 (± 0)	4.09 (± 0.07)	0 (± 0)	0 (± 0)	9.66 (± 0.04)	0 (± 0)	0 (± 0)	0 (± 0)	0 (± 0)	0 (± 0)	0 (± 0)	6.05 (± 0)	30.15 (± 0.65)	75.77 (± 1.23)
PC	0 (± 0)	0 (± 0)	8.29 (± 0.57)	0 (± 0)	0 (± 0)	23.70 (± 0.57)	0 (± 0)	0 (± 0)	36.81 (± 1.67)	0 (± 0)	0 (± 0)	2.59 (± 0.02)	0 (± 0)	0 (± 0)	24.10 (± 1.4)
PA	0 (± 0)	0 (± 0)	0 (± 0)	0 (± 0)	0 (± 0)	11.73 (± 0.57)	0 (± 0)	0 (± 0)	10.23 (± 0.57)	0 (± 0)	0 (± 0)	0 (± 0)	4.95 (± 0.05)	19.98 (± 1.67)	20.45 (± 1.57)
SA	0 (± 0)	0 (± 0)	22.06 (± 1.12)	0 (± 0)	0 (± 0)	44.78 (± 1.12)	0 (± 0)	0 (± 0)	41.53 (± 1.57)	7.65 (± 0.15)	35.82 (± 1.44)	36.48 (± 1.22)	25.43 (± 1.57)	60.01 (± 1.67)	64.66 (± 1.24)

Data are reported as percent of inhibition respect to the control (for which the inhibition was assumed = 0). Results are the mean of three experiments (± SD). EC: *E. coli*; LM: *L. monocytogenes*; PC: *P. carotovorum*; PA: *P. aeruginosa*; SA: *S. aureus.* ****p < 0.0001 compared with values obtained with EO (ANOVA followed by Dunnett’s multiple comparison test)

At zero time, the addition of the myrtle EO determined, at the highest concentration, a biofilm-inhibitory action, ranging between 26.25% (against *E. coli*) up to 66.67% (against *P. aeruginosa*). Interestingly, at the lowest concentration (0.4 mg/ml) the oil was practically ineffective against *P. carotovorum*, *P. aeruginosa* and showed very low effectiveness against *L. monocytogenes* and *E. coli*.

The influence exerted by the single components on the whole EO *activity* seemed different according to the bacterial species. Thus, α-pinene and linalool showed an inhibitory biofilm activity at 2 mg/ml, equal to 20.76% and 33.68%, respectively. On the other hand, the inhibitory biofilm action of the EO against *L. monocytogenes* could be due to α-pinene and myrtenyl acetate.

All EO components were active against *P. carotovorum,* except myrtenyl acetate, which showed an inhibitory biofilm activity only of 2.08%. On the contrary, the four main components resulted both in blocking or limiting the formation of the biofilm by *P. aeruginosa* and *S. aureus*. In the latter case, α-pinene determined an inhibitory efficacy of 91.39%.

The addition of the EO after 24 hours did not determine an effective inhibition of the biofilms. *E. coli* was completely insensitive to myrtle EO. Similar behavior was observed in the case of *P. aeruginosa* (inhibitory efficacy = 4.97%), *P. carotovorum* (inhibitory efficacy = 2.8%), and *S. aureus* (inhibitory efficacy of only 1.1%). Only *L. monocytogenes* still seemed, albeit weakly, sensitive to the presence of the myrtle EO (15.99%).

*E*. *coli* was completely insensitive to the presence of linalool and α-pinene (which instead were effective when added at zero time). *L*. *monocytogenes* seemed influenced by α-pinene (efficacy = 28.98%) and linalool, but not by myrtenyl acetate as at time zero. The addition of EO main components the 24 h mature *P. aeruginosa* biofilm had a low inhibitory efficacy ranging from 6.33% for myrtenyl acetate to 16.61% for 1,8 cineole. The best inhibitory efficacy on the 24 h mature *S. aureus* biofilm was showed by myrtenyl acetate (inhibitory efficacy = 50.62 % at 2 mg/ml).

The activity of the EO against the 48 h bacterial biofilms revealed a still different situation. An efficacy boost of the EO, which acted against *E. coli* with an inhibitory efficacy of 42.49% was observed. Only *S. aureus* returned to be sensitive to the action of the whole EO, albeit with a low efficacy (22.06%), but the four main components showed much more efficacy ranging between 36.48% (α-pinene) and 64.66% (linalool). Also, against 48 h biofilm of *L. monocytogenes* the EO was less active (4.09%), than linalool that exhibited, if tested alone, a strong inhibition (75.77%).

A similar setting was observed with regard to *P. carotovorum* on which the EO exhibited an efficacy of 8.29%, although three of the four main components showed an efficacy ranging between 23.70% (myrtenyl acetate) and 36.81% (1.8 cineole) against this ultra-mature biofilm.

*P. aeruginosa* was completely insensitive to the action of the EO, although some of the main components were capable of acting even partially on the 48 h biofilms.

All these data seem to indicate that the inhibitory efficacy was very strong if the EO was added at zero time, with a strong inhibition of biofilm formation. When the EO was added to 24 h biofilm, its effectiveness decreased drastically and increased again in 48 h biofilm.

A different scenario appeared when the effects of EO myrtle and its main components were evaluated on the metabolism of microbial cells, in the same at zero 24h and 48 h times, namely in the absence and in the presence of a mature or ultra-mature biofilm, respectively. The results are shown in Table 4.

**Table 4:** Capability of *M. communis* EO and its main components to inhibit the biofilm metabolic activity

Time	Myrtle EO (mg/ml)			Myrtenyl acetate (mg/ml)			1,8-Cineole (mg/ml)			α-Pinene (mg/ml)			Linalool (mg/ml)		
0	**0.4**	**1**	**2**	**0.4**	**1**	**2**	**0.4**	**1**	**2**	**0.4**	**1**	**2**	**0.4**	**1**	**2**
EC	21.49 (± 0.57)	27.15 (± 1.33)	37.59 (± 0.57)	0 (± 0)	0 (± 0)	61.34 (± 1.67)^****^	0 (± 0)	0 (± 0)	0 (± 0.0)	0 (± 0)	6.19 (± 0.15)	33.75 (± 1.33)	0 (± 0)	0 (± 0)	56.72 (± 1.67)^****^
LM	6.81 (± 0.57)	38.74 (± 0.57)	68.06 (1.57)	20.05 (± 2.05)^****^	39.25 (± 3.32)	41.73 (± 2.05)	0 (± 0)	39.62 (± 4.56)	55.17 (± 1.33)	27.72 (± 1.67)^****^	29.83 (± 0.57)	67.71 (± 2.33)	33.42 (± 1.67)^****^	43.42 (± 0.57)	54.47 (± 3.67)
PC	23.56 (± 1.33)	57.93 (± 1.67)	60.82 (± 1.67)	46.18 (± 1.07)^****^	59.10 (± 2.33)	60.77 (± 2.57)	20.38 (± 2.33)	30.44 (± 4.21)	35.65 (± 2.33)	0 (± 0)	0 (± 0)	36.81 (± 1.67)	0 (± 0)	62.31 (± 3.33)	77.84 (± 2.67)^****^
PA	0 (± 0)	0 (± 0)	50.19 (± 1.57)	95.12 (± 1.33)^****^	95.72 (± 0.57)^****^	96.99 (± 0.57)^****^	0 (± 0)	0 (± 0)	0 (± 0)	0 (± 0)	31.32 (± 1.67)^****^	40.22 (± 1.33)	30.12 (± 1.33)^****^	48.54 (± 2.57) ^****^	59.59 (± 3.33) ^***^
SA	0 (± 0)	0 (± 0)	0 (± 0)	0 (± 0)	50.29 (± 1.67)^****^	78.13 (± 1.67)^****^	0 (± 0)	5.76 (± 0.57)^*^	12.03 (± 0.57)^***^	76.23 (± 1.67)^****^	78.07 (± 1.67)^****^	89.41 (± 1.67)^****^	0 (± 0)	72.34 (± 3.56)^****^	77.05 (± 4.87)^****^
24 h	**0.4**	**1**	**2**	**0.4**	**1**	**2**	**0.4**	**1**	**2**	**0.4**	**1**	**2**	**0.4**	**1**	**2**
EC	0 (± 0)	0 (± 0)	6.36 (± 1.57)	0 (± 0)	0 (± 0)	32.29 (± 1.67)	0 (± 0)	0 (± 0)	0 (± 0.0)	0 (± 0)	0 (± 0)	16.62 (± 1.33)	0 (± 0)	0 (± 0)	0 (± 0)
LM	24.44 (± 1.57)	61.65 (± 2.67)	68.65 (± 1.57)	0 (± 0)	0 (± 0)	50.89 (± 2.67)	0 (± 0)	0 (± 0)	2.59 (± 1.67)	22.56 (± 1.67)	42.80 (± 2.25)	43.73 (± 2.33)	0 (± 0)	0 (± 0)	0 (± 0)
PC	0 (± 0)	0 (± 0)	64.11 (± 1.67)	36.28 (± 1.67)	52.70 (± 2.25)	68.70 (± 2.25)	0 (± 0)	0 (± 0)	0 (± 0)	35.75 (± 1.25)	63.27 (± 1.67)	71.59 (± 1.67)	27.86 (± 0)	45.04 (± 4.34)	45.20 (± 2.34)
PA	13.43 (± 1.67)	32.86 (± 2.24)	45.44 (± 1.57)	37.77 (± 1.73)	53.62 (± 1.57)	70.79 (± 1.67)	9.65 (± 0.15)	20.35 (± 0.65)	52.79 (± 2.21)	11.21 (± 0.86)	30.31 (± 1.57)	65.31 (± 1.67)	0 (± 0)	0 (± 0)	38.10 (± 0.57)
SA	31.86 (± 1.67)	51.10 (± 1.45)	65.30 (± 2.25)	34.52 (± 0.57)	63.39 (± 1.67)	69.77 (± 1.57)	0 (± 0)	0 (± 0)	0 (± 0)	11.33 (± 1.33)	26.77 (± 2.67)	35.88 (± 1.57)	17.98 (± 0)	37.44 (± 1.57)	44.92 (± 3.67)
48 h	**0.4**	**1**	**2**	**0.4**	**1**	**2**	**0.4**	**1**	**2**	**0.4**	**1**	**2**	**0.4**	**1**	**2**
EC	0 (± 0)	0 (± 0)	0 (± 0)	0 (± 0)	0 (± 0)	26.53 (± 1.67)	0 (± 0)	0 (± 0)	31.08 (± 0.0)	3.44 (± 0)	10.23 (± 0.15)	45.84 (± 1.33)	0 (± 0)	5.14 (± 0)	8.77 (± 0.57)
LM	0 (± 0)	0 (± 0)	60.63 (1.57)	17.74 (± 2.06)	39.17 (± 3.32)	49.97 (± 2.05)	0 (± 0)	0 (± 0)	53.92 (± 1.33)	25.67 (± 1.67)	45.49 (± 0.57)	61.83 (± 2.33)	0 (± 0)	4.19 (± 0.57)	55.51 (± 3.67)
PC	0 (± 0)	0 (± 0)	0 (± 0)	0 (± 0)	0 (± 0)	0 (± 0)	4.09 (± 2.33)	14.07 (± 4.21)	38.28 (± 2.33)	0 (± 0)	0 (± 0)	0 (± 0)	0 (± 0)	0 (± 3.33)	24.18 (± 1.67)
PA	0 (± 0)	0 (± 0)	23.04 (± 1.57)	0 (± 0)	0 (± 0)	15.73 (± 0.57)	0 (± 0)	0 (± 0)	43.89 (± 0)	20.56 (± 0)	40.30 (± 1.67)	40.71 (± 1.33)	0 (± 0)	0 (± 0)	14.51 (± 1.13)
SA	0 (± 0)	0 (± 0)	44.96 (± 0)	0 (± 0)	0 (± 0)	7.37 (± 0.57)	12.44 (± 0)	31.36 (± 0.57)^*^	32.19 (± 0.57)	0 (± 0)	0 (± 0)	32.19 (± 1.21)	0 (± 0)	0 (± 0)	0 (± 0)

The test was performed with 0.4, 1 and 2 mg/ml that were added at time zero, on 24 h-biofilms and on 48 h-biofilms, using the MTT assay. Results are reported as percent of inhibition respect to the control (for which the inhibition was assumed = 0). Results are the mean of three experiments (± SD). EC: *E. coli*; LM: *L. monocytogenes*; PC: *P. carotovorum*; PA: *P. aeruginosa*; SA: *S. aureus*. * *p* < 0.05;*** *p* < 0.001; ****p < 0.0001 compared with values obtained with EO (ANOVA followed by Dunnett’s multiple comparisontest)

The addition of the EO at time zero determined a different behavior on bacterial cells. It was effective against four of the five microorganisms tested and, at the highest concentration, the inhibitory effect on cellular metabolism ranged between 37.59% (*E. coli*) and 68.06% (*L. monocytogenes*). *S. aureus* was the only resistant strain.

In *E. coli*, myrtenyl acetate and linalool had a good activity (metabolic inhibition = 61.34% instead 1,8 cineole resulted completely ineffective.

As regards *L. monocytogenes*, it could be hypothesized that the bacterial metabolism was influenced by all four components, which showed an inhibitory efficacy ranging between 41.73% (myrtenyl acetate) and 67.71% (α-pinene). α-Pinene (36.81%) and 1,8 cineole (35.65 %) had lower efficacy than EO vs *P. carotovorum*; in fact, the EO showed an inhibitory efficacy on microbial metabolism of 60.82%. The influence of the myrtenyl acetate against *P. aeruginosa* resulted in a good inhibitory metabolic activity (96.99%). Instead, 1,8 cineole was completely ineffective. Instead, against the metabolism of *L. monocytogenes* 24 h biofilm the more active compounds were myrtenyl acetate (50.89%) and α-pinene (43.73%). For *P. carotovorum*, all components inhibited its metabolism, except 1,8 cineole, which was completely ineffective.

The 24 h biofilm of *S. aureus* was very sensitive to the EO (65.30%) and to the presence of myrtenyl acetate (69.77%) more than to the presence of the other 3 components, whose inhibitory efficacy did not go beyond 44.92%.

The addition of the samples on ultra-mature 48 h biofilms showed, once again, a different behavior. In this case, *S. aureus* continued to be influenced by the presence of the whole EO that, although to a lesser extent, with inhibition of 44.96%. Once again, the EO proved effective against *L. monocytogenes*, inhibiting the cellular metabolism by 60.83% (with a loss of about 8% compared to the previous condition).

This means that, in the case of *S. aureus*, the action of the whole EO seemed to be mainly influenced by the status of the biofilm, and that the EO acted more effectively on mature (24 h) ultra-mature (48 h) biofilms. In the case of *L. monocytogenes*, the EO seemed to have no "preferences", acting in all three times in a similar way, albeit slightly decreasing on ultra-mature biofilms.

The EO resulted completely ineffective against *P. carotovorum* and its inhibitory efficacy against metabolism of *P. aeruginosa* progressively decreased from a mature biofilm to an ultra-mature biofilm.

### Cytotoxic activity

**Fig. 2:**
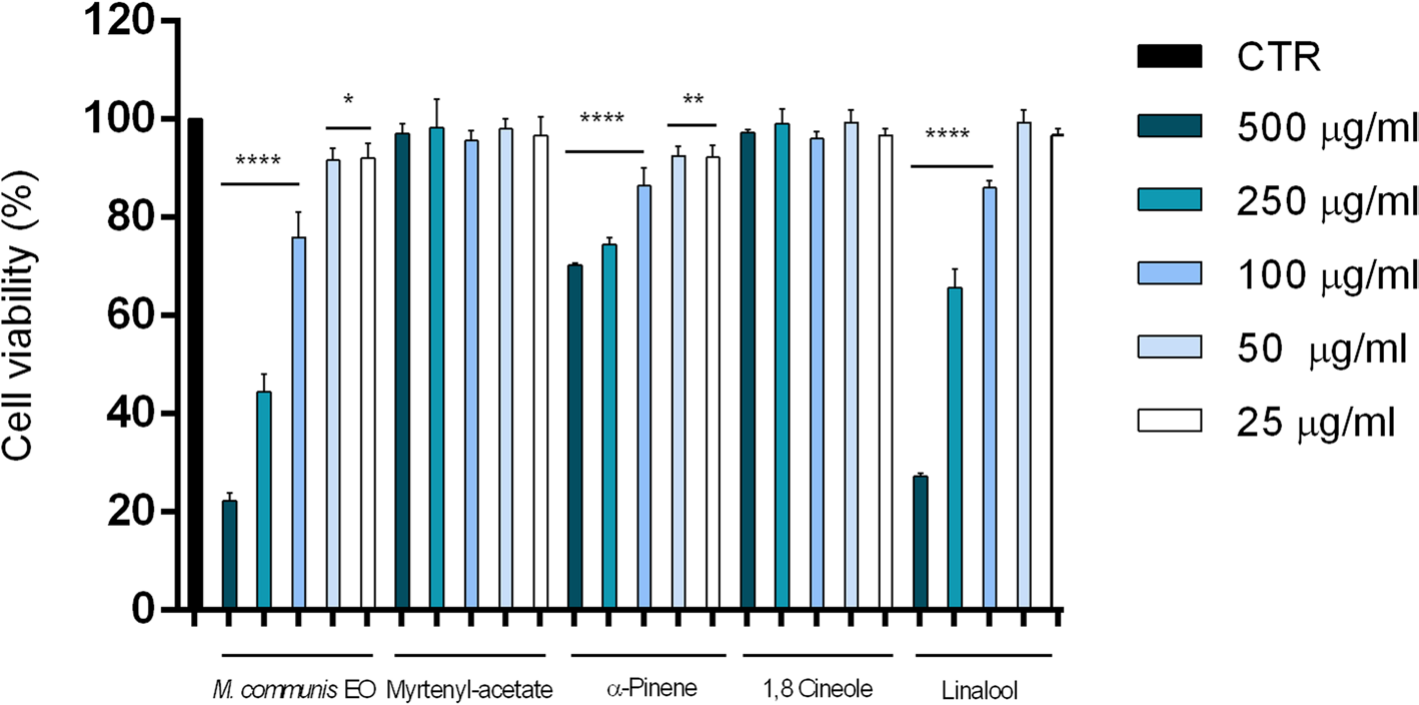
Cell viability calculated as percentage after MTT assay. Cells were treated with different concentrations (500–25 μg/ml) of *M. communis* essential oil, myrtenyl-acetate, α-pinene, 1,8 cineole and linalool for 24 h and solvent (DMSO, 0.1%) alone. Data are the mean ± SD of three experiments *p < 0.05, ** *p* < 0.01, **** *p* < 0.0001 vs. DMSO

The cytotoxicity of *M*. *communis* EO, myrtenyl acetate, α-pinene, 1,8 cineole, and linalool was evaluated using an MTT assay performed on the human neuroblastoma cell line (SH-SY5Y). After 24 h of treatment, the essential oil and its main constituents revealed very different cytotoxic activities, as reported in Fig. 2.

*M*. *communis* essential oil and α- pinene showed significant cytotoxicity at all concentrations tested even if their IC_50_ values are very different from each other; in fact, IC_50_ of essential oil was 209.1 μg/ml and for α- pinene was > 3 mg/ml. Linalool had significant activity against SH-SY5Y cells at concentrations ranging from 100 to 500 μg/ml, and showed an IC_50_= 356.3 μg/ml. Instead, myrtenyl-acetate had not cytotoxic activities with an IC_50_ > 2 g/ml. Treatment of SH-SY5Y neuroblastoma cells with the EO for 24 h resulted in a stronger cytotoxic activity respect to its principal constituents; these results suggested a synergic action between the EO components.

### Anti-acetylcholinesterase activity

**Fig. 3:**
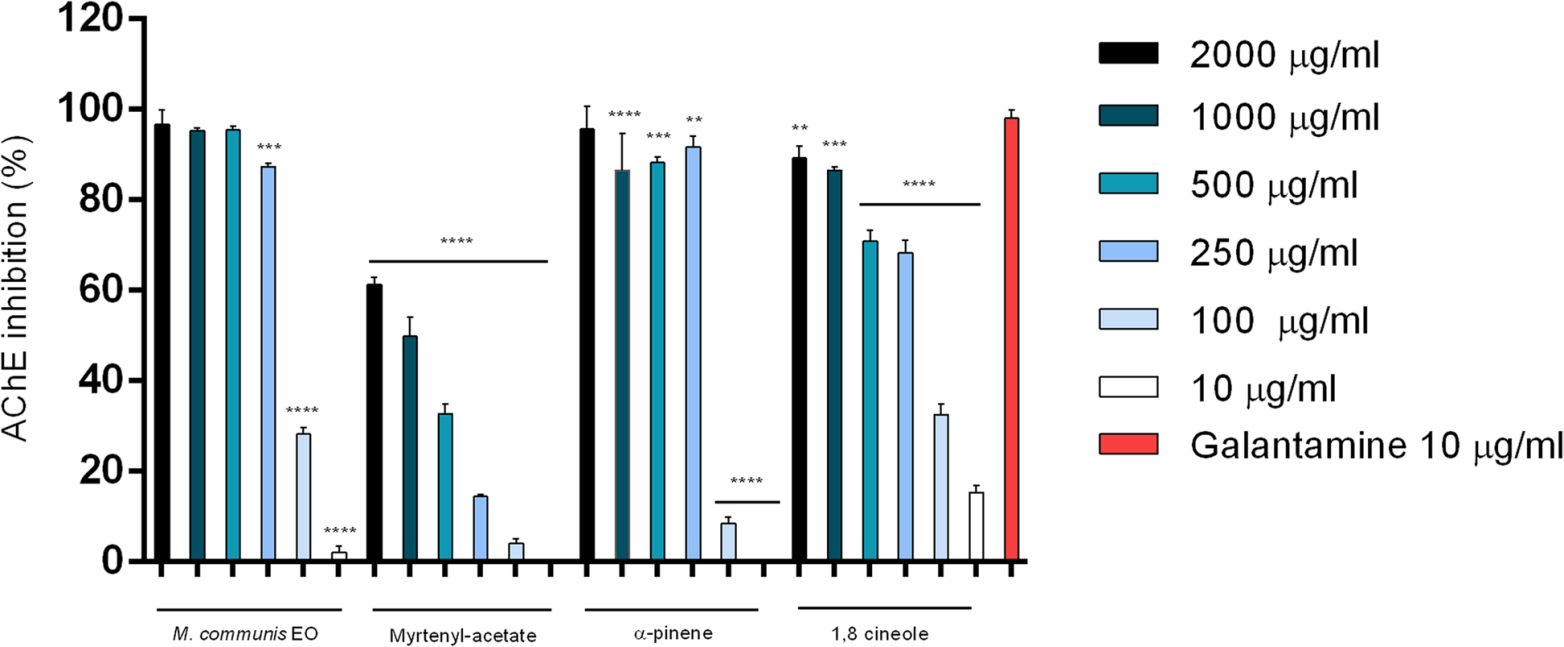
Dose-dependent inhibitory activity of *M. communis* EO, myrtenyl-acetate, α-pinene and 1,8 cineole against AChE. Data are given as mean ± SD (*n* = 3). *p < 0.05, ** *p* < 0.01, *** *p* < 0.001, **** *p* < 0.0001 vs. Galantamine

The acetylcholinesterase inhibitory activity of *M. communis* EO and its main constituents was evaluated by Ellman’s spectrophotometric method. Except for linalool, all tested substances showed AChE inhibitory activity and were able to inhibit in vitro the enzyme in a concentration-dependent manner (Fig. 3).

The IC_50_ values are reported in Table 5. 1,8-Cineole and α-pinene exhibited the most promising activities with IC_50_ values of 13.5 and 15.0 µg/ml, respectively. Interesting activity against AChE was also observed for the whole EO (IC_50_ = 32.8 µg/ml), whereas a weak AChE inhibitory activity was found for myrtenyl acetate (IC_50_ = 111.4 µg/ml).

**Table 5:** Acetylcholinesterase inhibitory activity of *M. communis* leaves essential oil and its main constituents

Sample	IC_50_ (μg/ml)
*M. communis* EO	32.8 ± 2.1
Myrtenyl-acetate	111.4 ± 7.3
1,8 Cineole	13.5 ± 1.5
α-Pinene	15.0 ± 1.0
Linalool	> 200
Galantamine (positive control)	0.7 ± 0.3

IC_50_ values are the mean ± SD of three experiments (*n* = 3)

Anyway, the activities of myrtle EO and its main constituents are good if compared with galantamine, which showed an IC_50_ of 0.7 μg/ml.

## Discussion

### Phytochemical analyses

Bradesi and coworkers hypothesized two chemotypes of *M. communis*, according to the presence/absence of myrtenyl acetate [39]. Our sample seems to belong to the first chemotype because it was characterized by a high content of this compound (29.80%).

Our results agree with Boelens and Jimenez, who reported the chemical composition of a Spanish myrtle essential oil with a high percent of myrtenyl-acetate (>30.0%) and a lower content of α-pinene (< 8.50%) [39]. Moreover, in the EO from an Algerian wild myrtle, myrtenyl-acetate (38.7%), 1,8-cineole (12.7%), and α-pinene (13.7%) were the main components [40]

Chalchat and coworkers studied the EOs of *M. communis* from different regions of the Mediterranean area and showed that in the essential oils from Tunisia and Corsica, α-pinene (51.2-52.9%; 53.5-56.7%), 1,8-cineole (24.1-24.7%; 18.8-21.3%) and limonene (6.1-7.3%; 5.0-5.2%) were the main constituents while myrtenyl-acetate was present in little amounts (0.1-0.3%; 0.8%); instead 1,8-cineole (32.5-37.5%) and myrtenyl-acetate (14.8-21.1%) were the principal constituents in the Moroccan and coast of Montenegro EOs [3, 41].Another myrtle EO from Tunisia showed two predominant components 1,8 cineole-55.09% and α-pinene 33.14%, and myrtenyl acetate was absent [42–44].Badra and coworkers reported limonene (33.4%) as the principal constituents of the EO from a myrtle sample collected in northeastern Algeria; this component was also present in different amounts in all previously cited studies but not in our sample [45].

Italian samples of EO showed many differences between regions. Two myrtle EOs from two locations of Liguria (Italy) presented α-pinene as the principal constituent (41.6% and 28.9%, respectively); myrtenyl-acetate and myrtenol were absent [45]. Moreover, the EOs from of 52 genotypes of *M. communis* growing in the same collection field at Oristano (Sardinia, Italy) showed α-pinene, limonene, 1,8-cineole, α-terpineol, and linalool as main components with few differences among samples [46–49]; in our sample limonene was absent and α-terpineol present in low percentage (2.1%). These results suggested that the Sardinian myrtle EOs belong to the α-pinene, 1,8-cineole, limonene chemotype and are characterized by the lack of myrtenyl acetate. Instead, in nine samples of EOs from *M. communis* leaves myrtenyl acetate was present as the main or second main compound depending on chemotype [50].

The chemical composition of myrtle essential oil is highly variable due to several factors such as growing conditions (climate, altitude, humidity, temperature, etc.), geographical position, and season or vegetative period of the plant [51]. Moreover, a close link exists among light-shade conditions, essential oil yield, and morphological parameters [52].

### Antibacterial activity

The results of different antibacterial activities highlighted that the chemical composition of the EO was ineffective or weakly effective against the tested Gram-negative bacteria. At the same time, it acted more strongly on the metabolism of the tested Gram-positive, albeit with different efficacy, according to the microorganism [18].

The data obtained suggested that myrtle EO seemed to be very effective *ab origine* on the biofilm formation if added at zero time, while its effectiveness has practically been canceled by adding it to mature biofilms (after 24 hours of growth). The addition of the EO to ultra-mature biofilms (after 48 hours of growth) seemed to increase again the effectiveness of EO myrtle, which was particularly effective against *E. coli*. When it was unable to act against the formation of the biofilm, the EO proved, however, effective by inhibiting bacterial metabolism in mature and ultra-mature biofilms, demonstrating that this EO was able to act both on young biofilms and mature and ultra-mature biofilms (through the action on bacterial cellular metabolism).

*M. communis* EO is known for its antimicrobial activity, generally ascribed to its chemical composition rich in monoterpene hydrocarbons and oxygenated monoterpenes such as linalool, carvacrol, α-pinene, and 1,8-cineole [53]. Like Berka-Zougali and coworkers [54], we observed a wide spectrum of action of myrtle EO. Moreover, other researchers reported a weak antibacterial activity of the EO against *S*. *aureus* and *E*. *coli*, due to the presence of its major components, such as 1,8-cineole and α-pinene [55]. Few studies are available in the literature on the ability of the myrtle EO to inhibit the formation of biofilms by pathogens [56–58]. Cannas and coworkers reported its efficacy in inhibiting biofilm formation by different *Candida* species, such as *C. albicans*, *C. parapsilosis*, and *C*. *tropicalis* [59]. Moreover, an isopropyl acetate extract obtained from myrtle leaves has proven effective in limiting the formation of biofilms by *Propionibacterium acnes*, also by acting on already mature biofilms.

The biofilm formation is responsible for several consequences, such as the production of exopolysaccharides, swimming, and swarming motility [60, 61]. Extracts and essential oils from several medicinal plants have been exploited as antibiofilm agents for pathogenic biofilm forming bacteria and fungi. They offer a virtually large and sustainable resource of very interesting classes of biologically active compounds. The prevention and/or control of biofilms by plant derivatives can occur through one or several mechanisms affecting the structure or the metabolism of the bacterial cells. There is a huge trend in relation to the identification of natural products that could possess anti-biofilm activity. myrtenol is a component of myrtle essential oil, demonstrated antibiofilm activity against *S. aureus* [62]. Myrtle EO was proved to block the metalloproteinase matrix activity [63], a mechanism involved in biofilm formation, as demonstrated by Xsia Tay and coworkers [64]. It has been used in combination with the EOs of *Alchemilla vulgaris* and *Eucalyptus* sp., to limit the formation of biofilms by *Peptostreptococcus stomatis* [65]. It was also shown inhibitory activity against *S. mutans*, *S. sanguinis*, and *S. salivarius*, therefore with a potential field of applicability in infections of teeth and oral cavity [66]. It is possible to hypothesize that myrtle EO and its main constituents analyzed in the present research could operate, depending on the strain, damaging the bacterial cell wall functions, or negatively affecting bacterial metabolism and enzymatic processes [60]. The myrtle EO may represent a product with a broad power against the pathogenic species *E. coli*, *P. aeruginosa*, *L. monocytogenes*, and *S. aureus*, resulting in particular scientific and practical interest due to the increased number of microbial species showing resistance to antibiotics [18, 53]. It is also important to highlight the activity of the EO against the Gram-negative *P. carotovorum*, known mainly as an agro-food pathogen infecting some of the most common crops such as potato, pineapple, and maize [58]. As is known, once the "niches" of biofilm, containing cells and other material (nucleic acids, proteins, polysaccharides) are formed, the cells present inside the biofilm tend to modify its metabolic pathways, not only to become more resistant than the homologous planktonic cells but also to express a greater "virulence". This means that starting from this step, it is more difficult to eradicate a possible infection using synthetic antibiotics, conventionally effective against the corresponding planktonic cells [60, 67]. In our study, the myrtle EO was able both to inhibit the viability of the cells present to the inner of biofilm, and/or to modulate the metabolic pathway that leads to greater bacterial virulence. The myrtle EO and its main components were studied in the antibacterial activity of these pathogens during the life of a biofilm, from immature to ultra-mature, associating the biofilm formation test (carried out with violet crystal) with an assessment of the biofilm metabolic activity (evaluated using the MTT reduction assay). This test has been used to evaluate the cytotoxicity of the myrtle EO in antimalarial assays [68] and to evaluate some biological properties, including the cytotoxicity of EOs from different areas of Algeria [69], Morocco [21], or Saudi Arabia [70]. The study performed by Alves and coworkers on thyme EOs demonstrated their ability to inhibit the biofilm formation and their influence on the viability of the cells entrapped within the biofilm, so to avoid or limit the subsequent cell changes occurring in [71]. The myrtle EO has been tested to inhibit the bacterial quorum-sensing mechanism [72]. The broad range of activity lead to incorporate it into chitosan edible films as an effective and safe strategy to deliver the oil to the foods [73]. In our experiments, the presence of myrtle EO, distinctly reduced the metabolic activity of cells in biofilms after 48 h of incubation. Our data also provided a preliminary indication that myrtle EO could affect the metabolic activity, as previously reported for the EO of *Rosmarinus officinalis*, rich in 1,8 cineole and α-pinene [74].

### Cytotoxic activity

Few studies reported the potential cytotoxic activity of *M. communis* EO on cancer cell lines [20–22] and no one on SH-SY5Y cells. However, our sample was more active (IC_50_ = 209.1 μg/ml) than myrtle EOs reported in the literature. Scazzocchio et al. showed no toxicity of a commercial myrtle EO on HeLa cells after a 24 h treatment [20]; Harassi and coworkers reported moderate cytotoxicity of two Moroccan myrtle EOs against MCF7 and P815 cells, with IC_50_ ranging from 4.0 to 6.25 μg/ml for MCF7 and from 53.9 to 260 μg/ml for P815 cells after 48 h [21]. On HT29 cell line, *M. communis* EO from Yemen reached after 72 h an IC50 of 110 μg/ml [22]. Anyway, our IC_50_ value was > 20 μg/ml indicating that the essential oil was not cytotoxic as judged by the criterion set by the National Cancer Institute that stated that only natural substances with IC_50_ < 20 μg/ml were considered to be cytotoxic against the treated cells [75].

### Anti-acetylcholinesterase activity

Only one study has been reported on the possible neuroprotective effects of *M. communis* EO. Sicilian EOs from the leaves of *Myrtus communis* L. stored in a collection orchard located at the experimental station ‘Orleans’ of the University of Palermo (Italy) showed lower acetylcholinesterase inhibitory activity than our sample, with IC_50_ values ranging from 96.0 to 520.2 μg/ml [27]. Another study regarding different myrtle leaf extracts displayed a moderate AChE inhibitory activity of *M. communis* [76].

No studies are available in the literature concerning the possible inhibitory AChE activity for myrtenyl acetate and linalool. Instead, our results confirm the good inhibitory activity for α pinene and 1,8 cineole. In fact, Dohi and coworkers reported IC_50_ values of 0.022 and 0.015 mg/ml, respectively [26].

Our findings revealed that the inhibitory activity of the whole EO results from a synergistic activity between the constituents. Further studies will be carried out to determine the possibility to use myrtle EO and/or its main constituents as coadjutant in the treatment of neurological disease.

## Conclusions

This study provides a phytochemical profile for the EO from the leaves of *M. communis* from Cilento area, never investigated before. Moreover, the effects of this EO on the biofilm formation and biofilm cells metabolic activity, the cytotoxicity on SH-SY5Y cells and its possible activity as an anti-acetylcholinesterase inhibitor were evaluated. Results could open new perspectives for the application of *M. communis* EO as the potentialproduct against the resistant pathogenic species *E. coli, P. aeruginosa, L. monocytogenes*, and *S. aureus*. Moreover, the results obtained with cytotoxicity on SH-SY5Y cells used as a model of neuronal cells and the good activities as acetylcholinesterase inhibitors made myrtle EO and its main constituents candidates for further studies on their possible use as coadjutants in the treatment of neurological diseases.

## Data Availability

The datasets used and/or analysed during the current study are available from the corresponding author on reasonable request.

## Abbreviations

AChE: Acetylcholinesterase
AChI: Acetylthiocholine iodide
DMSO: Dimethyl sulfoxide
DNTB: 5,5-Dithio-bis-(2-nitrobenzoic acid)
EO: Essential oil
FBS: Foetal bovine serum
FID: Flame ionization detector
GC: Gas chromatography
IC: Inhibitory concentration
MIC: Minimum inhibitory concentrations
MS: Mass spectrometry
MTT: 3-(4,5-Dimethylthiazol-2-yl)-2,5-Diphenyltetrazolium Bromide
OD: Optical density
PBS: Phosphate buffered saline
RPMI: Roswell Park Memorial Institute
